# The Evolving Landscape of Host Biomarkers for Diagnosis and Monitoring of Tuberculosis

**DOI:** 10.3390/biomedicines13092076

**Published:** 2025-08-26

**Authors:** Yang Cui, Haoran Li, Tianhui Liu, Rujie Zhong, Jiaying Guo, Jian Du, Yu Pang

**Affiliations:** Department of Bacteriology and Immunology, Beijing Chest Hospital, Beijing Tuberculosis and Thoracic Tumor Research Institute, Capital Medical University, Postal No 9, Beiguan Street, Tongzhou District, Beijing 101149, China

**Keywords:** tuberculosis, host-derived biomarkers, diagnosis

## Abstract

Tuberculosis (TB) remains a formidable global public health challenge. The rising prevalence of drug-resistant TB and increased human immunodeficiency virus(HIV) co-infection further exacerbate TB control efforts. Mycobacterium tuberculosis (Mtb) achieves highly heterogeneous infection outcomes (active disease, latency, or clearance) through immune evasion and host metabolic reprogramming. While conventional diagnostic techniques offer cost-effectiveness and accessibility without complex infrastructure, they are constrained by low sensitivity, prolonged turnaround times, and an inability to distinguish latent TB infection (LTBI) from active TB disease (ATB). Recent research into host-derived biomarkers provides a promising strategy to overcome diagnostic bottlenecks by deciphering characteristic molecular changes in host–pathogen interactions. This review systematically reviews advances in host-derived biomarkers for TB diagnosis, critically discussing the clinical potential, translational challenges, and future research directions of integrated multi-omics biomarker panels to enhance diagnostic sensitivity and specificity, differentiate ATB from LTBI, and guide precision therapy.

## 1. Introduction

Tuberculosis (TB), a chronic infectious disease caused by Mycobacterium tuberculosis (Mtb), continues to pose a significant threat to global public health. According to the latest World Health Organization (WHO) data, an estimated 10.8 million new TB cases and 1.25 million deaths occurred globally in 2023. Furthermore, the emergence of multidrug-resistant TB (MDR-TB) and extensively drug-resistant TB (XDR-TB) significantly complicates TB management. China, bearing a high TB burden, reported an estimated 741,000 new TB cases and 29,000 drug-resistant cases in 2023. Alarmingly, only approximately 20% of drug-resistant TB patients received standardized treatment, underscoring how delayed resistance diagnosis intensifies TB control challenges [1].

Mtb, due to its complex pathogenic mechanism, causes different immune responses after infecting the host. In immunocompetent hosts, Mtb exploits immune evasion mechanisms to establish persistent latency, resulting in latent TB infection (LTBI) [2]. Globally, approximately one-quarter of the population harbors LTBI, of whom 5–10% progress to active TB disease (ATB) due to waning immunity (e.g., HIV infection, diabetes, or organ transplantation), leading to pulmonary necrosis, cavity formation, and disseminated infection. Critically, autoimmune diseases (e.g., diabetes), cancer, and associated immunosuppressive therapies significantly elevate LTBI reactivation risk through compromised immune control, while concurrently, chronic inflammation from LTBI may exacerbate autoimmune conditions and oncogenesis [2,3,4].

Current TB diagnosis primarily relies on direct pathogen detection. Sputum smear microscopy with acid-fast bacilli (AFB) staining, the most widely used clinical method, requires a bacillary load ≥10^4^ CFU/mL for detection and exhibits an unacceptably high false-negative rate (up to 50%) [5]. While Mtb culture remains the diagnostic gold standard, its prolonged turnaround time (3–6 weeks) critically delays treatment initiation [6]. Immunological assays like the tuberculin skin test (TST) and interferon-gamma release assays (IGRAs) detect LTBI but fail to differentiate it from active disease. Their accuracy is also compromised by prior BCG vaccination and cross-reactivity with non-tuberculous mycobacteria (NTM) [7,8]. Molecular tests like GeneXpert Mtb/RIF, which detect Mtb DNA and rifampicin resistance-conferring mutations (rpoB gene), reduce diagnosis time to 2 h. However, their sensitivity declines significantly in extrapulmonary TB, pediatric patients, and HIV-coinfected individuals [9,10]. In recent years, artificial intelligence has demonstrated breakthrough potential in multi-omics integration. Its capabilities extend not only to high-throughput data analysis but also to uncovering the combinatorial diagnostic value of low-abundance biomarkers, which is crucial for enhancing confidence in tuberculosis differentiation [11].

**Figure 1:**
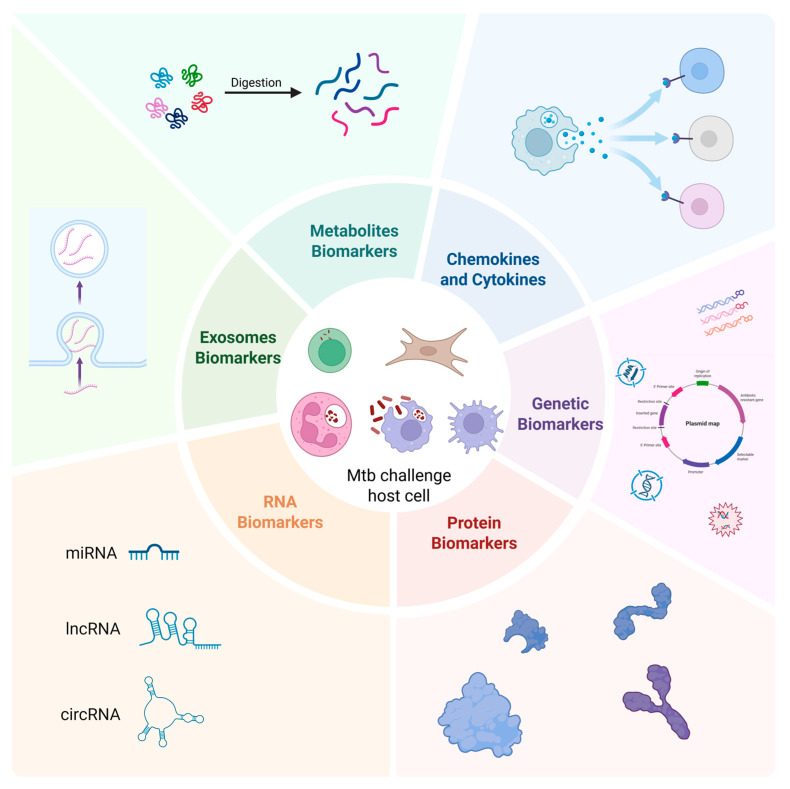
Schematic overview of host-derived biomarkers for tuberculosis diagnosis and monitoring.

To address the above phenomena, the research focuses on the host immune response and metabolic remodeling triggered by Mtb infection. Host-derived biomarkers reflecting molecular signatures of pathogen–host interplay offer novel strategies to overcome traditional diagnostic constraints. Compared to pathogen-directed approaches, host biomarkers provide key advantages, including the following: (1) host immune responses activate rapidly post-infection, preceding detectable pathogen replication, enabling early diagnosis [12]; (2) distinct molecular signatures differentiate LTBI and ATB, permitting infection staging [13]; (3) reduced dependence on sample type or bacillary load enhances utility in pediatric and HIV-coinfected populations [14]; (4) dynamic biomarker monitoring facilitates treatment response assessment [15]. This review systematically examines novel diagnostic technologies based on host-derived biomarkers. It provides an in-depth discussion of integrated multi-omics biomarker panel strategies, their clinical potential for improving diagnostic accuracy, differentiating ATB from LTBI, guiding precision treatment, current translational bottlenecks, and future research priorities (Figure 1).

## 2. Genetic Biomarkers

Genetic biomarkers are increasingly recognized as precision medicine tools for rapidly diagnosing cancer and genetic disorders, offering advantages like non-invasive detection and treatment optimization in screening and personalized therapy [16,17]. In TB diagnostics, host genetic biomarker research demonstrates multi-dimensional utility, extending beyond ATB/LTBI differentiation to sex-specific diagnosis, immunosuppressed state identification, and treatment monitoring [18,19].

Studies reveal significant sex-based differences in pediatric TB gene expression signatures (Table 1). In males, *slamf8*, *gbp2*, *wars*, and *fcgr1c* expression distinguishes TB from healthy controls (HC) with 85% sensitivity and 70% specificity. In females, a panel comprising *gbp6*, *celsr3*, *aldh1a1*, and *gbp4* achieves comparable diagnostic efficacy (85% sensitivity, 69% specificity), approaching WHO target product profiles (TPP) for non-sputum-based tests [20,21]. These findings underscore the importance of sex-specific factors in pediatric TB diagnosis. However, the male and female signatures identified in three African cohorts require validation in other populations.

Single-cell RNA sequencing identified significantly upregulated ADM expression in myeloid cells of ATB patients, distinguishing ATB from HC (AUC = 0.89). Its regulatory network (hsa-miR-24-3p-NEAT1-ADM-CEBPB axis) elucidates key TB immunomodulation mechanisms [22]. Guanylate-binding protein (GBP) family genes show diagnostic utility across TB presentations; gbp5 effectively diagnoses pulmonary TB (AUC = 0.88) and HIV-coinfected TB meningitis (AUC = 0.86) [23]. Genes including *gbp5*, *batf2*, and CD64 are significantly overexpressed in ATB patient peripheral blood mononuclear cells (PBMCs), enabling LTBI differentiation (AUC = 0.879, 0.911, and 0.85, respectively) [24,26]; *gbp5*, *bnip3*, *klf6*, *dysf*, *LASP1*, and *pcbp1* are significantly upregulated in the plasma cfRNA of active tuberculosis patients [27]. Conversely, *klf2* and *znf296* downregulation in LTBI distinguishes LTBI from HC, identifying 49% of IGRA false-negative LTBI individuals. Combined analysis suggests low *klf2* and *znf296* expression in LTBI correlates with immune dysregulation, potentially serving as sensitive IGRA adjuncts [28]. Aberrant *rac1*, *rbx1*, *mrpl33*, and *elavl1* expression in Mtb-infected THP-1 cells indicates potential for predicting drug resistance [29].

Multi-modal diagnostic strategies enhance clinical utility. Komakech et al. evaluated the performance of a point-of-care (POC) triage test based on a tri-gene panel (*gbp5*, *dusp3*, *klf2*). While the area under the curve (AUC) of 0.79 (79% sensitivity, 85% specificity) fell short of the WHO target product profile (TPP) for a triage test (≥90% sensitivity, ≥70% specificity), it performed significantly in terms of both C-reactive protein (CRP; AUC 0.68) and the monocyte-to-lymphocyte ratio (MLR; AUC 0.63) [21,25]. Further investigations revealed downregulation of metabolic reprogramming markers (e.g., *fhit*, *man1c*) in active tuberculosis (ATB) patients. A combined diagnostic model incorporating these markers achieved an AUC of 0.87 for distinguishing between ATB and LTBI [30]. Conversely, the glycosyltransferase *b4galt5* and *kcnj2* were found to be upregulated in TB patients. Remarkably, a combination model utilizing these markers achieved an exceptionally high AUC of 0.979. Mechanistic insights suggest these markers regulate apoptotic pathways via a competing endogenous RNA (ceRNA) network, underscoring the translational value of multi-marker synergistic diagnostic approaches [31]. Building upon this, Chang et al. [27] identified significant upregulation of *gbp5*, *bnip3*, *klf6*, *dysf*, *lasp1*, and *pcbp1* in the cell-free RNA (cfRNA) of active TB patients’ plasma. Notably, *gbp5* expression correlated positively with mycobacterial load (r = 0.757). A diagnostic signature based on these six genes demonstrated high performance in discriminating TB-positive from TB-negative individuals. The validation cohort achieved an AUC of 0.95, with 97.1% sensitivity and 85.2% specificity. Crucially, this high diagnostic accuracy (AUC > 0.90) was maintained across diverse geographical regions and HIV statuses, surpassing the performance of signatures derived from whole blood RNA. This six-gene cfRNA signature meets the optimal criteria outlined in the WHO TPP for a non-sputum-based triage test [21,27].

Combining the myeloid-specific ADM biomarker with Xpert Ultra significantly improves diagnostic sensitivity (AUC > 0.85) [22]. Dynamic monitoring reveals that a high subpopulation frequency of macrophage *rgs1* correlates strongly with treatment response in osteoarticular TB (OTB). Post-treatment restoration of this subpopulation differentiates OTB from HC and bacterial bone infections (AUC > 0.85), aiding rare TB diagnosis [32]. For HIV-coinfected individuals, *gbp5* diagnostic performance improves under immunodeficiency (AUC: From 0.74 to 0.86), while the *gbp5*, *dusp3*, and *klf2* combination maintains specificity via stable epigenetic modifications, offering an adapted solution for this high-risk group [23,28].

Mechanistic insights uncover core TB regulatory networks. Weighted gene co-expression network analysis (WGCNA) identified the ADM-IFIT3-SERPING1 panel, whose diagnostic performance surpasses single markers [23]. Furthermore, the SP110 rs9061 polymorphism is associated with increased LTBI risk, likely by reducing plasma TNF-α levels and weakening immune control, highlighting genomic markers’ value in complementing transcriptomic analysis [33]. Single-cell sequencing elucidates cellular heterogeneity: ADM overexpression occurs primarily in TB myeloid cells [23], while the macrophage-specific *rgs1*high subpopulation influences treatment response via autophagy-related gene *atg5* regulation [32]. These discoveries underpin cell-type-targeted precision diagnostics.

Promising genetic biomarkers include ADM, the combination of *b4galt5*/*kcnj2* and the six-gene combination signature panel including *gbp5*, *bnip3*, and *znf296*, given their high diagnostic performance (AUC > 0.85) and mechanistic relevance. Sex-specific genes (e.g., *slamf8*, *gbp6*) and epigenetic marks (e.g., SP110 SNP) offer new directions for specialized populations. Dynamic host anti-TB immunity drives gene expression alterations linked to immune mechanisms. Further exploration of genes like *gbp5*, *ADM*, *znf296*, *klf2*, and *rgs1*, investigating their roles in immune regulation (e.g., NF-κB inhibition, myeloid activation), transcriptional repression (e.g., *znf296*/*klf2* downregulation), macrophage subpopulation imbalance (e.g., *rgs1*high-ferroptosis link), and pathogen evasion, coupled with multi-cohort validation, will strengthen their application in precision TB diagnosis, treatment monitoring, and development of host-directed therapy.

## 3. RNA Biomarkers

Non-coding RNAs, particularly long non-coding RNAs (lncRNAs) and circular RNAs (circRNAs), function as pivotal hubs in dynamic networks governing pathogen recognition, immune modulation, and disease outcome [34,35,36]. Their tissue-specific expression, stability in body fluids, and disease association position them to overcome traditional diagnostics’ sensitivity and specificity limitations [37].

LncRNAs exert regulatory control in TB by modulating epigenetic phenomena (chromatin remodeling, histone modifications, DNA methylation), immune gene expression, and signaling pathways [38]. Their tissue-specific and infection-stage-dependent expression provides sensitive markers for disease state differentiation (Table 2). LncRNA-miRNA interactions expand their value in the immune networks field [39]. Research has identified significantly elevated LINC00152 and LARS2-AS1 levels in ATB patients and infected macrophages, distinguishing ATB from LTBI [40]. Additionally, downregulation (PCED1B-AS1) in ATB patient PBMCs suggests potential as an early diagnostic marker [41]. High-throughput sequencing revealed increased NEAT1 expression in TB patients, normalizing post-treatment, indicating prognostic utility [42]. Deep learning models based on m6A-associated lncRNAs (e.g., LINC00460, LINC01116) achieved 92% accuracy (AUC = 0.935) in diagnosing HIV/TB co-infection, demonstrating AI’s power in multi-omics integration [43]. Furthermore, combining NR_038221 and NR_003142 to regulate immune pathways via competitive endogenous RNA (ceRNA) networks, significantly enhanced TB/HC discrimination, with NR_038221 showing the strongest correlation [44]. Lung tissue-specific lncRNAs (e.g., ENST00000497872, n333737) are differentially expressed in smear-negative TB patients, correlating with lesion metabolic activity, thereby offering new insights for non-invasive diagnosis [45].

MiRNAs play central roles in Mtb immune evasion and inflammation by targeting mRNA stability/translation. Their dynamic, tissue-specific expression allows precise discrimination between active Infection and latency, changing significantly during therapy [53]. Stability and detectability in body fluids make miRNAs ideal non-invasive targets. The miR-29 family (e.g., miR-29a) is significantly elevated in ATB patient serum/sputum, correlating positively with disease activity (AUC = 0.87) and functioning as a key ATB/LTBI discriminator by regulating T-cell apoptosis and monocyte function [46]. Notably, miR-31 is considered among the most promising diagnostic markers, with sensitivity (96%) and specificity (89%) approaching WHO TPP minimum targets, particularly excelling in pediatric TB [21,54]. Moreover, miR-4433 b-5p and miR-424-5p expression patterns partially address DR-TB diagnosis challenges, offering molecular targets via drug metabolism pathway influence [12]. Combinatorial biomarkers enhance precision; a model with hsa-let-7d-5p and hsa-miR-140-5p excelled in LTBI/ATB differentiation (training AUC = 0.930, sensitivity 100%, specificity 88.5%; test AUC = 0.923, sensitivity 100%, specificity 92.3%) [47]. In HIV/TB co-infection, combining miR-223-5p and miR-10 b-5p, which regulate Mtb growth and pro-inflammatory cytokines (e.g., IL-6, IL-8), achieved AUC = 0.96 (sensitivity 86.7%, specificity 91.7%), providing a specific co-infection marker [48].

CircRNAs exhibit superior degradation resistance in body fluids due to their covalently closed structure and nuclease resistance, suiting long-term monitoring and resource-limited settings [55]. Studies show significant hsa_circ_001937 upregulation in PBMCs (AUC = 0.873) and hsa_circRNA_103571 downregulation in plasma (AUC = 0.838) of ATB patients, both distinguishing ATB from HC [49,50]. DR-TB diagnosis advanced significantly, with the circRNA_051239 elevation in patient serum achieving AUC=0.9738, highlighting the drug resistance marker potential [51].

Combinatorial strategies boost performance; hsa_circ_0001204 and hsa_circ_0001747 were used in combination and achieved AUC = 0.928 [52]. The triple panel (circRNA_029965, circRNA_051239, circRNA_404022) reached AUC = 0.992 (sensitivity 97.1%, specificity 85.2%), maintaining efficacy across regions/HIV status, meeting WHO standards [27].

Promising RNA biomarkers include miR-31, circRNA_051239, and LINC00152, due to their high sensitivity, stability, and mechanistic depth. Infection-induced host immunity changes drive RNA alterations intrinsically linked to immune mechanisms. For instance, miR-155 regulates macrophage autophagy/cytokine secretion in anti-TB immunity; its elevation in serum in ATB patients yielded 91% sensitivity. MiR-31 antagonizes immunosuppressive signals, showing high pediatric TB specificity (AUC = 0.96). CircRNAs (e.g., circRNA_051239) regulate resistance genes by sponging miRNAs like miR-320a; dynamic changes strongly correlate with sputum culture conversion (r = 0.76), enabling real-time treatment monitoring. LncRNAs (e.g., NEAT1, LINC00152) influence immune balance via Toll-like receptor pathways/T-cell differentiation, with aberrant expression linked to disease activity. However, lncRNA mechanisms require deeper investigation; e.g., NEAT1 influences Mtb survival by regulating macrophage apoptosis, but downstream targets and clinical translation need validation. These RNA changes reflect host immune status (e.g., M1/M2 polarization, CD8+ T-cell exhaustion) and directly correlate with Mtb evasion mechanisms (e.g., autophagy inhibition, inflammatory microenvironment remodeling). Further investigation into combinatorial RNA-based signatures, such as miR-155, circRNA_051239, and lncRNA NEAT1, along with cross-population validation of clusters like GBP5/KLF6, can enhance stratified diagnostic frameworks and immunotherapeutic approaches. Incorporating single-cell sequencing to delineate the spatiotemporal dynamics of granulomatous RNA expression and employing wearable sensors for real-time monitoring may mitigate static diagnostic constraints and foster the translation of precision medicine for tuberculosis.

## 4. Protein Biomarkers

Protein biomarkers offer high specificity/sensitivity, enabling early screening, precise classification, and dynamic monitoring. Non-invasive detection in body fluids and established use in oncology/cardiovascular/metabolic diagnostics underscore significant clinical value [56,57]. In TB, they have demonstrated breakthrough potential, enhancing diagnostic accuracy (AUC > 0.95) via high-sensitivity detection (>90%) and multi-dimensional signal integration (e.g., immune, metabolic pathways), overcoming limitations for smear-negative cases and LTBI detection [6,58].

Proteomics have driven recent TB diagnostic breakthroughs (Table 3). For fluid markers, serum Adenosine Deaminase 2 (ADA2) and CD14 combination exhibit significant diagnostic efficacy, distinguishing ATB from HC (AUC = 0.972, sensitivity 90.6%, specificity 90.0%), suiting resource-limited areas [59]. Sputum/saliva shows aberrant calcium-binding protein S100-A11, haptoglobin (HP), and complement C3 expression, reflecting pulmonary immune activation [60,61,62]. Immune checkpoint molecules have gained attention as novel biomarkers: Monocyte PD-L1 expression in ATB correlates positively with bacterial load and decreases post-treatment, indicating disease activity assessment and treatment monitoring value [63]. CTLA-4 dynamic changes on CD4^+^ T cells relate to immunoregulatory function; its post-treatment elevation offers new perspectives for LTBI differentiation [64].

Single-marker limitations drive combinatorial panel research. A triple marker panel (I-309, SYWC, kallistatin) achieved WHO TPP standards (sensitivity 90%, specificity 70%) for ATB/non-TB differentiation, outperforming C-reactive protein (CRP). The I-309/SYWC combination performed well in Peru/South Africa (AUC > 0.85) but less effectively in Vietnam, suggesting host genetics and pathogen strain differences impact generalizability [21,65]. A multinational study identified a five-protein panel (ANXA5, KRT6B, LCN2, ORM1, MMP8) differentiating ATB, LTBI, and HC with 84% overall accuracy (ATB: 97%, LTBI: 72%, HC: 79%), enabling precise stratification [28,66]. Excluding ATB samples revealed a six-protein panel (MCEMP1, HPX, SPRR2F, IGKV4-1, VDAC2, LMNA) distinguishing LTBI from HC with 97.7% accuracy, the most accurate reported LTBI vs. HC panel [66].

A six-protein panel (FETUB, FCGR3B, LRG1, SELL, CD14, ADA2) is significantly upregulated in ATB plasma; combined detection achieved AUC = 0.972 (sensitivity/specificity > 90%) [59]. Adenosine deaminase (ADA) activity markedly elevates in TB pleurisy pleural fluid, serving as a key diagnostic adjunct [67]. Acute-phase proteins Alpha-1-acid glycoprotein (AGP1) and Alpha-1-antitrypsin (ACT) are overexpressed in ATB patients, correlating with inflammation severity [68].

Proteins like S100-A9 and superoxide dismutase (SOD) are highly expressed in TB lesions, participating in oxidative stress/tissue repair; levels correlate with disease severity [69]. TIMP-2 and TSP4 influence progression by regulating granuloma extracellular matrix remodeling [70]. CD14 shows significant serum changes in HIV/TB co-infection, suggesting an association with immunosuppression association [72]. Novel strategies include ESAT6-CFP10-stimulated PID1 gene detection, achieving 100% TB/pneumonia differentiation [73].

Metabolism-related protein/inflammation marker studies demonstrate that Serum Amyloid A (SAA) (sensitivity 96.88%/specificity 78.43%) combined with lipid markers (HDL-C, Apolipoprotein A-I) achieved 96.88% sensitivity, particularly for AFB-negative cases [71].

Promising protein biomarkers include immune checkpoint molecules (PD-1/PD-L1), the six-protein panels (FETUB et al.) and (MCEMP1 et al.) [59,66], which have high diagnostic performance, precise classification, and age applicability. Biomarker panels and detection protocols require tailoring for TB types/populations. Host anti-TB immunity changes trigger protein alterations linked to immunoregulatory mechanisms. For example, immune activation significantly upregulates PD-1/PD-L1 signaling on CD8^+^ T cells/granuloma microenvironments, synergizing with IDO-1 to create immunosuppressive niches; deeper mechanistic investigation and cross-cohort validation will enhance disease activity/treatment response assessment. Aberrant metabolism-related protein expression (e.g., ORM1, RBP4) reveals host lipid dysregulation-immunosuppression interactions, elucidating ORM1-mediated immunosuppressive pathways and pathogen survival roles, which could underpin metabolic intervention-diagnostic strategies. Sex-specific marker differential expression (e.g., SLAMF8, GBP6) highlights immunological sex heterogeneity; exploring sex-related regulatory network impacts will inform personalized diagnostic tool design. Systematic elucidation of protein biomarker immune mechanism associations and robust multi-region/population validation will accelerate TB diagnostic innovation and translation.

## 5. Chemokine and Cytokine Biomarkers

Cytokines are small signaling proteins secreted by immune/tissue cells (e.g., IL, IFN, TNF). As core intercellular communication mediators, they regulate immune responses (e.g., Th1/Th2 balance), inflammation (e.g., IL-6 pro-inflammatory effects), hematopoiesis, and tissue repair via autocrine, paracrine, or endocrine actions [74]. In disease, they drive pathology (e.g., TNF-α in rheumatoid arthritis) and can serve as therapeutic targets (e.g., anti-TNF mAbs) or diagnostic markers (e.g., CRP) [75,76].

Significant progress elucidates host chemokine/cytokine application and mechanisms in TB diagnosis/stratification. Chemokines centrally enable TB typing/drug resistance discrimination by dynamically reflecting immune response intensity. Cytokine expression profile changes reflect TB immune status/pathology (Table 4). CXCL9 (MIG) and CXCL10 (IP-10) are significantly upregulated in ATB patients, with levels correlating positively with disease activity. Studies demonstrate high diagnostic efficacy for distinguishing drug-susceptible (DS-TB) from drug-resistant TB (DR-TB) (AUC = 0.84 and 0.82, respectively). Notably, CXCL9 and CXCL10 showed perfect diagnostic performance (AUC = 1.00) for DR-TB vs. HC, highlighting unique values for precise DR-TB stratification [77]. IP-10, an IFN-γ-inducible protein, performs robustly among chemokines; standalone sensitivity/specificity rivals IGRA, suiting paucibacillary sample screening [78]. IP-10 + IFN-γ increased sensitivity by 12%, though reduced CRP efficacy in HIV co-infection requires consideration [79]. IP-10 showed 92% sensitivity/85% specificity in pediatric ATB, though LTBI/ATB differentiation remains controversial across studies, potentially due to age/infection stage heterogeneity [80]. Significantly reduced IL-21 and IP-10 levels in HIV/TB co-infection suggest immunosuppression markers, offering new co-infection diagnostic perspectives [81]. CXCL1 excels in DS-TB/LTBI differentiation (AUC = 0.85), suggesting potential for early infection screening [82]. Based on a Chinese cohort study, the chemokine CCL8 demonstrated exceptional performance as a single host biomarker in distinguishing active tuberculosis (ATB) from latent tuberculosis infection (LTBI). The validation cohort showed a specificity of 100% and a sensitivity of 90.8% (AUC = 0.988). Combined with CXCL9, the overall diagnostic performance was further enhanced (AUC = 0.958) [83].

IFN-γ and TNF-α elevation in ATB patients correlates with bacillary load/inflammatory damage [87]. Serum cytokines/inflammatory mediators (especially MMP-2, OPN, BAFF, and multiprotein combinations) show significant potential in diagnosing childhood tuberculosis, effectively distinguishing infection status and providing new directions for developing childhood-specific TB diagnostic tools [85]. Post-treatment TNF-α decline inversely correlates with sputum culture conversion time, indicating a treatment monitoring value [88].

Multi-omics integration unlocks chemokine diagnostic potential. A combined model of lipid metabolite FAHFAs (e.g., FAHFA 18:2) and IL-8 (AUC = 0.975), targeting PPARγ-mediated inflammation suppression, significantly outperformed single markers [86]. A machine learning-optimized CXCL9/CXCL10/CXCL1 triad achieved >90% accuracy for LTBI/DS-TB/DR-TB stratification, providing a molecular basis for individualized treatment [77].

CXCL9, CXCL10, and CXCL1 represent the most promising chemokine biomarkers due to high diagnostic performance (AUC = 0.84–1.00) and precise differentiation of drug-sensitive/resistant TB and latent Infection. In TB, chemokines orchestrate immune responses via multi-dimensional mechanisms. For example, IFN-γ enhances macrophage antimicrobial function via JAK-STAT1 activation and induces CXCL9/CXCL10 expression, recruiting CXCR3+ T cells to infection sites. CXCL1 activates neutrophil oxidative burst via CXCR2, but excessive release may exacerbate tissue damage. Sustained high CXCL9/CXCL10 in DR-TB may reflect T-cell exhaustion, while IL-21 deficiency in HIV co-infection could impair B-cell responses, promoting immune evasion. Furthermore, lipid metabolite downregulation (e.g., FAHFA) may disrupt macrophage anti-inflammatory phenotypes via PPARγ signaling. Future research must elucidate how spatiotemporally defined chemokine gradients regulate granuloma immune cell interactions, how metabolic reprogramming (e.g., mTORC1 signaling) influences chemokine secretion, and whether pathogen proteins (e.g., ESAT-6) suppress chemokine production via TLR/NF-κB pathways. These investigations will support precision immunotherapies targeting chemokine axes (e.g., nano-delivered antagonists and gene editing to remodel microenvironments).

## 6. Metabolites Biomarkers

Metabolites are small molecules (e.g., saccharides, lipids, amino acids, and organic acids) directly reflecting real-time cellular metabolic statesing real-time cellular metabolic states. In disease diagnosis, metabolite changes often precede genetic/protein abnormalities, revealing pathway dysregulation (e.g., the Warburg effect in cancer). Rapid detection (mass spectrometry/NMR), low cost, and non-invasive screening suitability (urine, breath) make them vital precision medicine biomarkers [89,90].

Host metabolites gain prominence in TB diagnosis/pathogenesis (Table 5). Metabolomic biomarkers achieve high-sensitivity diagnosis (AUC > 0.9) by capturing host metabolic dysregulation (e.g., tryptophan-kynurenine pathway abnormalities, lipid reprogramming). Serum kynurenine-to-tryptophan ratio (Kyn/Trp) significantly elevates in ATB patients (AUC = 0.91), demonstrating high sensitivity/specificity for ATB/HC differentiation [84]. Characteristic amino acid/lipid alterations occur in TB: ATB patients exhibit increased serum leucine (Leu)/valine (Val) but decreased citrulline (Cit)/glutamine (Gln), potentially linked to mTOR activation and urea cycle inhibition [91]. 9-OxoODE and eicosapentaenoic acid (EPA) are significantly downregulated in ATB plasma, correlating with inflammation intensity/lipid peroxidation field [92]. DR-TB patients show significant lysophosphatidylinositol (Lyso-PI) enrichment, suggesting roles in modulating bacterial membrane stability to promote resistance [11].

Metabolomics–AI integration identified high-accuracy panels: Albumin + 9-OxoODE differentiated smear-positive TB from HC (accuracy 83.33%); l-pyroglutamic acid (PGA) + secretin achieved 92.86% accuracy for smear-negative TB [92]. A multilayer perceptron neural network (MLP) incorporating 20 metabolites (e.g., tryptophan, cortisol) simultaneously differentiated ATB/LTBI/HC with 94.74% accuracy [93]. Urinary neopterine + diacetylspermine showed 90% sensitivity/85% specificity, offering a novel non-invasive strategy [97].

Metabolites modulate immune cell function in TB pathogenesis. Kynurenine, an IDO pathway product, inhibits T-cell proliferation and promotes Treg differentiation, exacerbating granuloma immunosuppression [84]. PD-L1 and IDO-1 co-express in TB granulomas, forming an immune evasion axis via tryptophan depletion/kynurenine generation; dynamic changes reflect disease activity [94]. Furthermore, 5-oxoproline reduction in ATB serum directly correlates with pulmonary tissue damage severity [95]. l-5-Oxoproline as a single marker exhibits excellent specificity (94%) but insufficient sensitivity (47%). It is recommended to use a combination of stearic acid (AUC 0.855), l-cysteine (AUC 0.827), and citric acid (AUC 0.848) to enhance diagnostic accuracy (all three have AUC > 0.8 and sensitivity > 70%) [96].

The tryptophan metabolic pathway (Kyn/Trp) and LysoPE represent the most promising metabolite biomarkers due to high diagnostic performance (AUC > 0.99) and broad applicability (covering smear-positive/negative TB, active/latent infection). MTB infection drives immune responses/pathology by remodeling the host metabolic network. For instance, IDO-mediated tryptophan metabolism produces kynurenine, promoting Treg differentiation via AhR activation (suppressing Th1 immunity) and potentially directly interfering with macrophage antibacterial autophagy. Downregulation of lipids like 9-OxoODE may suppress anti-inflammatory phenotypes via PPARγ signaling, worsening inflammatory damage. Moreover, Mtb releases cell wall components (e.g., lipoarabinomannan), mimicking host metabolic signals and hijacking mitochondrial oxidative phosphorylation to promote intracellular survival. Therefore, an in-depth investigation into how metabolic reprogramming regulates immune cell polarization via mTOR/AMPK pathways and how metabolome-transcriptome/proteome cross-talk affects granuloma microenvironment homeostasis will provide a theoretical basis for host-directed therapies targeting metabolic nodes (e.g., IDO inhibitors, lipid modulators).

## 7. Exosome Biomarkers

Exosomes are nanoscale vesicles (30–150 nm) secreted by cells, carrying molecular cargo (proteins, nucleic acids, lipids) derived from parent cells. Distributed in blood/saliva/urine, they reflect aberrant intercellular communication in disease (e.g., tumor immune evasion), offering non-invasiveness, high stability, and disease-tissue-specific signal enrichment. They apply to early cancer detection, microenvironment monitoring, and targeted drug delivery fields [98]. As novel TB biomarkers, exosomes leverage lipid bilayer protection of biomolecules, integrating host immune/pathogen information to enhance detection sensitivity (AUC > 0.9) significantly [99]. Their nucleic acid, protein, and lipid components exhibit unique diagnostic potential.

Exosomal miRNAs are core TB diagnostic biomarkers due to their high stability/disease specificity (Table 6). miR-20 b-5p is significantly downregulated in exosomes from Mtb-infected macrophages [100], while let-7c-5p, miR-27-3p, and miR-25-3p are upregulated [101], suggesting roles in modulating inflammation/autophagy affecting TB progression. Clinically, miR-185-5p and miR-423-5p are significantly elevated in TB patient plasma exosomes (86.7% sensitivity, 91.7% specificity) [102]. NEosomal miRNA profiles are disease-stage-specific (e.g., hsa-let-7c-5p/hsa-miR-1246 differential expression in ATB vs. LTBI), providing molecular disease state differentiation [101,103].

Exosomal circRNAs’ closed circular structure confers exceptional stability, suiting DR-TB diagnosis. circRNA_051239 is significantly upregulated in DR-TB serum exosomes (AUC = 0.974), potentially regulating resistance genes by sponging miR-320a [104]. Additionally, circRNA_0002419/circRNA_0007919 upregulate in TB lesions, while circRNA_0005521 downregulates; differential expression correlates with macrophage polarization/autophagy [105]. Combinatorial strategies enhance performance; combining exosomal circRNA_001937 (AUC = 0.873) with plasma-free circRNA markers improves ATB diagnostic specificity [49]. Downregulation of exosomal circRNAs (e.g., circRNA_0001380) in ATB offers new non-invasive targets [106].

Exosomal proteins encompass Mtb-specific antigens/host response proteins, providing unique diagnostic perspectives. Host proteins like CD36/C4BPA show aberrant ATB expression, potentially linked to immune evasion/excessive inflammation [107,108]. While exosomal proteins reflect pathogen burden/host response simultaneously, reliance on mass spectrometry limits its use in resource-limited settings.

Less studied exosomal lncRNAs/lipids show emerging diagnostic value. Lipids like phosphatidylserine (PS)/lipoarabinomannan (LAM) enrich TB exosomes, potentially promoting Mtb survival by modulating host lipid metabolism. These markers expand diagnostic dimensions, but low abundance and detection complexity remain barriers [109].

MiR-185-5p and miR-423-5p are the most translationally promising exosomal biomarkers, with high diagnostic performance (AUC > 0.85) and clinical validation. While IP-10 performs well in serology, its exosomal expression requires investigation. Future efforts require optimized isolation techniques, expanded validation (especially extrapulmonary TB/children), and multi-marker models (e.g., Hsp16.3 + miR-185-5p + circRNA) to enhance diagnostic performance. Mtb infection hijacks host exosome networks to reshape the immune response-pathogen survival balance. Infected macrophage exosomes carry pathogen proteins (e.g., KatG, GroES), potentially interfering with dendritic cell antigen presentation by mimicking host antigens. Concurrently, miRNA downregulation (e.g., miR-20 b-5p) enhances pro-inflammatory cytokine secretion by relieving TLR4 signaling inhibition, creating inflammation-immunosuppression coexistence. Exosomal circRNAs (e.g., circRNA_051239) may activate autophagy genes (e.g., ATG5) by sponging miR-150-5p, promoting pathogen clearance, but overexpression may induce mitochondrial stress, exacerbating damage. Furthermore, exosomal lipids (e.g., phosphatidylserine) bind TAM receptors, inducing Treg expansion/suppressing Th1 polarization, enabling persistent Mtb survival.

Therefore, further mechanistic exploration will be crucial to reveal how exosomes spatiotemporally regulate granuloma immune cell interactions (e.g., delivering miR-27-3p to inhibit macrophage apoptosis/maintain pathogen niche); whether pathogen proteins (e.g., ESAT-6) evade lysosomal degradation by hijacking exosome sorting; details of metabolic-immune cross-regulation (e.g., exosomal lipids suppressing mitochondrial oxidative phosphorylation via PPARγ, weakening antibacterial function); and engineered exosomes’ therapeutic potential (e.g., siRNA targeting Mtb resistance genes, IL-12 delivery to remodel microenvironment). Deciphering these will advance “double-edged sword” strategies, developing high-sensitivity diagnostics and exploring exosomes as therapeutic delivery vehicles.

## 8. Expectations

**Figure 2:**
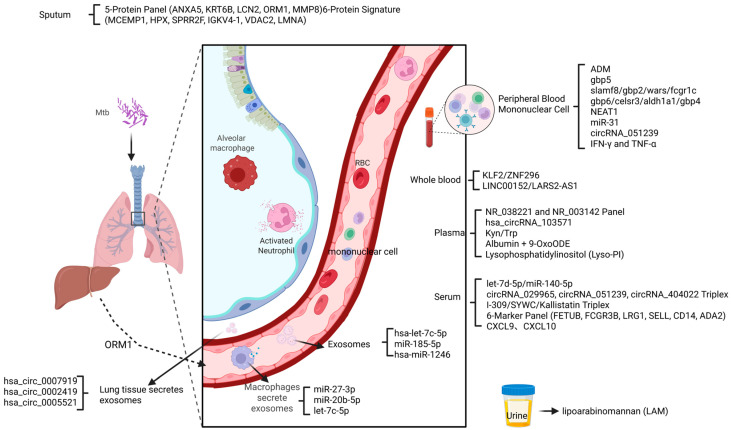
Multi-omics integration and technological advances in host biomarker research for tuberculosis.

In recent years, multi-omics technologies have driven tuberculosis host marker research breakthroughs (Figure 2). Genome-wide association analysis revealed that particular genetic markers can effectively diagnose active tuberculosis in Asia (89% specificity), but their cross-ethnic applicability needs to be validated. Transcriptomics revealed that miR-155 in combination with IP-10 protein and 5-oxoproline metabolite can differentiate between active and latent tuberculosis, which is significantly better than traditional tests. Integration of host miRNA and pathogen katG mutation signatures also significantly improved drug resistance diagnostic accuracy. The integration of innovative technologies such as single-cell sequencing, spatial transcriptomics, machine learning and exosome research has facilitated the screening of immunotherapeutic targets, the exploration of spatial heterogeneity of metabolic markers, and the reduction of assay costs and the expansion of its ubiquity.

However, clinical translation faces complex challenges. Population heterogeneity significantly affects the diagnostic efficacy of markers, and multicenter cross-ethnic cohorts are urgently needed to validate their broad applicability. Inadequate standardization and reproducibility of technologies are the core bottlenecks; differences in purity of exosome isolation methods (e.g., ultracentrifugation vs. kits) affect the consistency of downstream assays; heterogeneity of data from different metabolomics platforms (mass spectrometry vs. ELISA) makes it difficult to recognize each other’s results; and the lack of standardized operational protocols for the baseline expression of RNA markers (circRNAs, miRNAs) means processes are susceptible to interference by age, ethnicity, and sampling methods, exacerbating the barriers to comparability. The lack of standardization exacerbates the barrier to comparability. The lack of dynamic monitoring capability is another shortcoming, as the existing static markers are difficult to reflect the response to treatment or the risk of relapse in real time (e.g., CRP may be transiently elevated during the initial phase of treatment due to immune activation, making it impossible to distinguish between efficacy and deterioration). Furthermore, inadequate understanding of the mechanisms governing host biomarkers (e.g., CRP dynamics during treatment, miR-4433b-5p’s role in drug resistance) limits their precise application and integration. Unmet needs of special populations: metabolic markers for TB in children are disturbed by age fluctuations; HIV co-infection reduces the specificity of traditional markers; diagnosis of drug resistance still relies on genetic testing of pathogens, and insufficient integration of host immune profiles leads to a lag in treatment optimization. The imbalance between technology cost and accessibility also hinders adoption in resource-limited areas.

The future requires multi-dimensional breakthroughs. Integrating genes, RNA, proteins, metabolites and exosomes to build multimodal marker combinations is the key to improving performance, and machine learning can assist in screening the optimal combinations and modeling predictions. Integration of imaging and exosomal data is expected to establish an accurate typing framework and promote the integration of diagnosis, treatment and prognosis. The establishment of cross-platform standardized processes (e.g., consensus on exosome isolation, metabolite control guidelines) and multi-center validation will improve data comparability and translational efficiency. Specifically, future efforts will need to integrate AI-driven dynamic monitoring models to validate the cross-population applicability of low-abundance biomarker combinations (such as exosomal miR-185-5p + metabolite Kyn/Trp ratio) in HIV/pediatric tuberculosis, thus advancing targeted breakthroughs in precision diagnostic strategies for special populations, to contribute to the goal of ending tuberculosis by 2035.

## Figures and Tables

**Table 1:** Genetic biomarkers related information.

Biomarker	AUC	Sensitivity (%)	Specificity (%)	Cohort Characteristics	Sample	Reference
*slamf8* (↑), *gbp2* (↑), *wars* (↑), and *fcgr1c* (↑) Panel	0.86	85	73	Children with active TB (Male): Multicenter (Kenya, South Africa, Malawi), total n = 85 (TB:39; Other diseases:46) Includes HIV+/− subgroups.	Whole-blood transcriptome (PAX gene RNA tube)	[20]
*gbp6* (↑), *celsr3* (↓), *aldh1a1* (↑), and *gbp4* (↑) Panel	0.83	85	69	Children with active TB (Female): Multicenter (as above) Total n = 61 (TB:27; Other diseases:34) Includes HIV+/− subgroups.		
ADM (↑)	0.786–0.899	74–95	52–78	TB: 46 (Adult), 9 (Child) LTBI: 25, 9 HC: 37, 9 (Multicenter, China)	PBMC sc RNA-seq & Whole Blood	[22]
*gbp5* (↑) (single)	0.88	83	83	Multicenter (Uganda, Vietnam, Philippines); Adult PTB (n = 251, TB+ = 142)	Plasma cfRNA	[23]
*batf2*(↑)	-	-	-	ATB: 61; ORD/LTBI/HC: 143 (Single-center)	Whole blood	[24]
CD64 (↑)	-	-	-	ATB: 61; ORD/LTBI/HC: 143 (Single-center)	Whole blood	[24]
*gbp5/dusp3/klf2* Panel (↓)	0.9	83.3–90.7	59.8–75.6	LTBI: 24; HC: 37 (Single-center)	Whole blood	[25]

↑, Elevated expression; ↓, Upregulated expression;AUC, area under curve; TB, tuberculosis; ATB, active buberculosis; HC, healthy control; LTBI, latent tuberculosis infection; HIV, human immunodeficiency virus; ORD, operational research and demonstration.

**Table 2:** RNA biomarkers related information.

Biomarker	AUC	Sensitivity (%)	Specificity (%)	Cohort Characteristics	Sample	Reference
LINC00152 (↑)	0.919	76.36	72.73	LTB vs. ATB (55 LTB, 55 ATB); Single-center	Plasma	[40]
LARS2-AS1 (↑)	0.782	63.64	94.55	LTB vs. HC (55 LTB, 55 HC); Single-center	Plasma	[40]
LINC00152 (↑) + LARS2-AS1	0.829	68.18	83.64	LTB vs. HC (Combined model); Single-center	Plasma	[40]
m6A-modification-related lncRNAs (e.g., LINC00460, LINC01116)	0.935	92.9	90.9	HIV/TB vs. HIV (Training set: 14 HIV/TB, 11 HIV); Single-center	Whole blood	[43]
	0.904	93.8	80	HIV/TB vs. HIV (Validation set: 15 HIV/TB, 16 HIV)		
NR_038221 and NR_003142 Panel (↑)	0.845	79.2	75	Active TB (TB): 52; Healthy Controls (HC): 52; Cohort: TB vs. HC; Single-center (Shaoxing Sixth Hospital)	Plasma	[44]
NR_038221 (↑)	0.677	-	-	Active TB (TB): 52; Healthy Controls (HC): 52; Cohort: TB vs. HC; Single-center (Shaoxing Sixth Hospital)	Plasma	[44]
Lung tissue-specific lncRNAs (e.g., ENST00000497872, n333737) (↓)	0.89	86	82	Clinically diagnosed PTB (no micro evidence), microbiologically confirmed PTB, non-TB disease controls, healthy controls; n = 1764 total (Validation cohort: 97 Clin Dx PTB + 140 Non-TB)	PBMC	[45]
miR-29a (↓)	-	-	-	HIV/HCV co-infected patients (n = 121)	Serum	[46]
miR-29a (↑)	-	-	-	COVID-19 patients (n = 20)	PBMC	[46]
hsa-let-7d-5p and hsa-miR-140-5p Panel (↓)	0.930 (Train)	100	88.5 (Train)	Train/Val Set: ATB:29, LTBI:25, HC:30; Cohort: ATB vs. LTBI vs. HC	Serum	[47]
	0.923 (Val)	-	92.3(Val)			[47]
miR-223-5p and miR-10b-5p Panel	0.79	-	-	ATB (drug-sensitive) vs. HC (55 ATB, 24 HC); Single-center	Serum	[48]
hsa_circ_001937(↑)	0.873	85	77.5	40 TB vs. 40 HC (adult active TB); Single-center	PBMC	[49]
	0.85	72.2	90	115 TB vs. 90 HC (independent validation cohort)	PBMC	
hsa_circRNA_103571 (↓)	0.838			ATB vs. HC (32 ATB, 29 HC); Single-center	Plasma	[50]
circRNA_051239 (↑)	0.85	71.43	66.67	DR-TB vs. DS-TB; Single-center	Serum	[51]
hsa_circ_0001204 and hsa_circ_0001747 Panel	0.92	NP	NP	APTB patients vs. HC; Single-center	Plasma	[52]

↑, Elevated expression; ↓, Upregulated expression;AUC, area under curve; PTB, pulmonary tuberculosis; ATB, active tuberculosis; LTBI, latent tuberculosis infection; HC, healthy controls; DR-TB, drug-resistant tuberculosis; DS-TB, drug-sensitive tuberculosis; HIV, human immunodeficiency virus.

**Table 3:** Protein biomarkers related information.

Biomarker	AUC	Sensitivity (%)	Specificity(%)	Cohort Characteristics	Sample	Reference
Serum ADA2 + CD14 Panel	-	-	-	HIV+/HIV− TB patients (n = 209)	Serum	[59]
I-309/SYWC (↑)/Kallistatin Triplex (↓)	0.9	90	70	479 Adults (177 TB, 302 Non-TB); Multicenter (South Africa, Peru, Vietnam; Performance ↓ in Vietnam)	Serum	[65]
I-309/SYWC Panel (↑)	0.88	89	74	479 Adults (177 TB, 302 Non-TB); Multicenter (South Africa, Peru, Vietnam; Performance ↓ in Vietnam)	Serum	[65]
5-Protein Panel (ANXA5, KRT6B, LCN2, ORM1, MMP8)	MCC = 0.767	84	84	Mixed cohort: PTB (n = 31), LTBI (n = 25), Healthy (n = 19)	Sputum	[66]
6-Protein Signature (MCEMP1, HPX, SPRR2F, IGKV4-1, VDAC2, LMNA)	MCC = 0.954	97.7	97.7	LTBI (n = 25) vs. Healthy (n = 19)	Sputum	[66]
6-Marker Panel (FETUB, FCGR3B, LRG1, SELL, CD14, ADA2)	0.972	90.6	90	PTB vs. HC (UK MIMIC cohort, n = 62)	Serum	[59]
Adenosine Deaminase (ADA) (↑)	-	40–100	68–100	259 Adults with pleural effusion (incl. 41 TPE)	Pleural Fluid	[67]
AGP1 (↑)	0.816	63.5	91.8	PTB vs. LTBI (Training set, n = 169)	Plasma	[68]
ACT (↑)	0.835	68.2	92.9	PTB vs. LTBI (Training set, n = 169)	Plasma	[68]
ACT, AGP1 and CDH1 Panel	0.946	82.3	92.8	PTB vs. LTBI (Training set PTB =85, LTBI = 84)	Plasma	[68]
	0.989	96.5	95.8	PTB vs. HC (Training set PTB = 85, HC = 71)		
S100A9 (↑)	0.891	86	90	STB group vs. MTB/HC (81 STB, 80 MTB, 50 HC)	Plasma	[69]
SOD1 (↓)	0.525	79	32	STB group vs. MTB/HC (81 STB, 80 MTB, 50 HC)	Plasma	[69]
TIMP-2 + TSP4	0.878	75	87.5	TB treatment 8 weeks vs. Baseline (n = 39)	Serum	[70]
SAA (↑)	0.98	96.88	78.43	129 Symptomatic Adults (97 TB, 32 Non-TB); Brazil, Single-center	Plasma	[71]
HDL-C (↓)	0.84	75	72.16	129 Symptomatic Adults (97 TB, 32 Non-TB); Brazil, Single-center	Plasma	[71]

↑, Elevated expression; ↓, Upregulated expression; AUC, area under curve; PTB, pulmonary tuberculosis; ATB, active tuberculosis; LTBI, latent tuberculosis infection; HC, healthy control; HIV, human immunodeficiency virus.

**Table 4:** Chemokines and cytokines biomarkers related information.

Biomarker	AUC	Sensitivity (%)	Specificity (%)	Cohort Characteristics	Sample	Reference
CXCL10 (IP-10) (↑)	0.84 (DR-TB vs. DS-TB)	-	-	Indian Adults: DR-TB (n = 40), DS-TB (n = 40), LTB (n = 40), HC (n = 40); Single-center	Plasma	[77]
	-	77	94	Belgian Children: Active TB (n = 12), LTBI (n = 18), Uninfected (n = 17); Single-center (Discovery cohort)	PBMC supernatant	
CXCL9 (MIG) (↑)	0.82 (DR-TB vs. DS-TB)	-	-	Indian Adults: DR-TB (n = 40), DS-TB (n = 40), LTB (n = 40), HC (n = 40); LTBI vs. ATB (Beijing Chest Hospital cohort, n = 208)	Plasma	[77]
	> 0.9	97	100	Belgian children: Active TB (n = 12), LTBI (n = 18), Uninfected (n = 17); Single-center (Discovery cohort)	PBMC supernatant	
CCL8	0.89	90.79	100	ATB vs. LTBI (IGRA-positive) (Beijing Chest Hospital cohort, n = 208) Single-center	Plasma	[83]
CCL8 + CXCL9	0.958	96	84.37	ATB vs. LTBI (IGRA-positive) (Beijing Chest Hospital cohort, n = 208) Single-center	Plasma	[83]
CXCL1 (↑)	0.80 (DR-TB vs. LTB)	-	-	Indian Adults: DR-TB (n = 40), DS-TB (n = 40), LTBI(n = 40), HC (n = 40); Single-center	Plasma	[82]
IFN-γ and TNF-α (↑)	-	100	100	Belgian Children Discovery cohort (n = 47)	PBMC supernatant	[84]
	0.918	84	94	153 Adults (45 Active TB, 108 Non-active TB incl. 38 LTBI); Single-center prospective cohort	PBMC (stimulated)	
BAFF/TNFSF13B (↑)	0.809 (HC vs. TB)	-	-	216 Polish children (aged 1–17 years) who received BCG vaccination (TB, n = 15; LTBI, n = 50; HC, n = 151)	Serum	[85]
MMP-2 (↓)	0.848 (HC vs. TB)	-	-	216 Polish children (aged 1–17 years) who received BCG vaccination (TB, n = 15; LTBI, n = 50; HC, n = 151)	Serum	[85]
FAHFAs (e.g., FAHFA 18:2) (↓) + IL-8(↑) Model	0.9754	92.3	96	Adult PTB (MTB, n = 26) vs. HC (n = 26); Single-center (Shanghai Pulmonary Hospital)	Serum	[86]
CXCL9, CXCL10, CXCL1 Triplex (↑)	Overall AUC 0.80	-	-	Indian Adults: DR-TB (n = 40), DS-TB (n = 40), LTB (n = 40), HC (n = 40); Single-center	Plasma	[77]
CXCL9 (↑)	0.8876 (RGM)	-	-	Indian Adults: DR-TB (n = 40), DS-TB (n = 40), LTB (n = 40), HC (n = 40); Single-center	Serum	[77]
	0.9042 (SGM)					
CXCL10 (↑)	0.8649 (MTB)	-	-	Adult PTB (MTB, n = 26) vs. HC (n = 26); Single-center (Shanghai Pulmonary Hospital)	Serum	[77]
IFN-γ (↑)	0.8387 (MTB)	-	-	Adult PTB (MTB, n = 26) vs. HC (n = 26); Single-center (Shanghai Pulmonary Hospital)	Serum	[86]
IL-8 (↑)	0.9186 (MTB)	-	-	Adult PTB (MTB, n = 26) vs. HC (n = 26); Single-center (Shanghai Pulmonary Hospital)	Serum	[86]
FAHFA 18:2 (↓)	0.8708 (MTB)	-	-	Adult PTB (MTB, n = 26) vs. HC (n = 26); Single-center (Shanghai Pulmonary Hospital)	Serum	[86]
	0.9440 (RGM)	-	-			

↑, Elevated expression; ↓, Upregulated expression; AUC, Area Under Curve; PTB, Pulmonary Tuberculosis; ATB, Active Tuberculosis; LTBI, Latent Tuberculosis Infection; HC, Healthy Controls; DR-TB, Drug-Resistant Tuberculosis; DS-TB, Drug-Sensitive Tuberculosis; BCG, Bacillus Calmette-Guérin; HIV, Human Immunodeficiency Virus.

**Table 5:** Metabolites biomarkers related information.

Biomarker	AUC	Sensitivity (%)	Specificity (%)	Cohort Characteristics	Sample	Reference
Glutamine (Gln) (↓)	0.581	-	-	Polish children (TB:15, LTBI:52, NMP:20, HC:149)	QFT TB1 Supernatant	[91]
Citrulline (Cit) (↓)	0.848	82	88	17 TBPE vs. 17 MPE (Adults); Single-center	Pleural Fluid	[91]
Lysophosphatidylinositol (Lyso-PI) (18:0) (↑)	0.94	-	-	17 Active TB vs. 16 household contacts (Adults); Single-center	Plasma	[11]
Albumin + 9-OxoODE	0.83	80	86	27 SPPT vs. 36 Controls (Adults); Single-center	Plasma	[92]
l-Pyroglutamic acid (PGA) + Secretin	0.93	86	100	37 SNPT vs. 36 Controls (Adults); Single-center	Plasma	[92]
MLP Model (20 Metabolites)	0.95	100	100	27 SPPT, 37 SNPT, 36 Controls (3-class); Single-center	Plasma	[93]
PD-L1 + IDO-1(↑)	-	-	-	TB patient granuloma tissue	Granuloma Tissue	[94]
5-Oxoproline (↑)	0.7	-	-	Discovery cohort: Haitian Active TB (n = 102) vs. HC (n = 102)	Serum	[95]
5-Oxoproline (l-5-Oxoproline) (↓)	0.709	47	94	17 TBPE vs. 17 MPE (Adults); Single-center	Pleural Fluid	[96]

↑, Elevated expression; ↓, Upregulated expression; AUC, area under curve; PTB, pulmonary tuberculosis; ATB, active tuberculosis; LTBI, latent tuberculosis infection; HC, healthy control; SNPT, Smear-negative pulmonary tuberculosis; SPPT, smear-positive pulmonary tuberculosis; TBPE, tuberculous pleural effusion; MPE, malignant pleural effusion; NMP, non-tuberculous mycobacterial pulmonary disease.

**Table 6:** Exosome biomarkers related information.

Biomarker	AUC	Sensitivity (%)	Specificity (%)	Cohort Characteristics	Sample	Reference
miR-20b-5p (↓)	-	-	-	RAW 264.7 macrophages (in vitro infection model)	Macrophages	[99]
hsa-let-7c-5p (↑)	-	-	-	Adult TB (n = 60), LTBI (n = 60), HC (n = 60)	Serum exosomes	[100]
mmu-miR-27-3p (↑)	-	-	-	Murine RAW264.7 macrophages (BCG infection model)	Macrophage exosomes	[100]
mmu-miR-25-3p (↑)	-	-	-	Murine RAW264.7 macrophages (BCG infection model)	Macrophage exosomes	[100]
miR-185-5p (↑)	0.75	50	93.75	20 Active TB Adults vs. 17 Healthy; Single-center	Plasma exosomes	[101]
hsa-miR-1246	-	-	-	Adult TB (n = 60), LTBI (n = 60), HC (n = 60)	Serum exosomes	[102]
mmu-let-7c-5p (↑)	-	-	-	Murine RAW264.7 macrophages (BCG infection model)	Macrophage exosomes	[100]
circRNA_051239 (↑)	0.9738	-	-	Active TB (n = 128) vs. CAP (n = 50) vs. HC (n = 50)	Serum	[103]
hsa_circ_0007919 (↑)	-	-	-	PTB lung tissue samples (n = 9 patients)	Lung tissue	[104]
hsa_circ_0002419 (↓)	-	-	-	PTB lung tissue samples (n = 9 patients)	Lung tissue	[104]
hsa_circ_0005521 (↓)	-	-	-	PTB lung tissue samples (n = 9 patients)	Lung tissue	[104]

↑, Elevated expression; ↓, Upregulated expression; AUC, area under curve; PTB, pulmonary tuberculosis; ATB, active tuberculosis; LTBI, latent tuberculosis infection; HC, healthy control; BCG, Bacillus Calmette-Guérin; CAP, community-acquired pneumonia; HIV, human immunodeficiency virus.

## Data Availability

The data presented in this study are available from the corresponding author.
